# Case report: Successful combination therapy with double-filtration plasmapheresis and rituximab under the condition of the use of a sensor-augmented pump for type B insulin resistance syndrome

**DOI:** 10.3389/fendo.2022.997296

**Published:** 2022-09-09

**Authors:** Arata Osanami, Masatoshi Kanda, Tatsuya Sato, Chikako Akazawa, Shuhei Baba, Hiroaki Komatsu, Kazuyuki Murase, Tomohisa Yamashita, Toshiyuki Yano

**Affiliations:** ^1^ Department of Cardiovascular, Renal and Metabolic Medicine, Sapporo Medical University School of Medicine, Sapporo, Japan; ^2^ Department of Rheumatology and Clinical Immunology, Sapporo Medical University School of Medicine, Sapporo, Japan; ^3^ Department of Cellular Physiology and Signal Transduction, Sapporo Medical University School of Medicine, Sapporo, Japan; ^4^ Department of Medical Oncology, Sapporo Medical University School of Medicine, Sapporo, Japan

**Keywords:** type B insulin resistance (TBIR), double-filtration plasma apheresis, rituximab, sensor-augmented pump (SAP), ketosis, catabolism, systemic lupus erythematosus, Sjögren’s syndrome

## Abstract

Type B insulin resistance syndrome (TBIR) is a rare disease characterized by refractory diabetes due to severe insulin resistance caused by anti-insulin receptor autoantibodies, and a standard treatment regimen for TBIR has not been established, leading to therapeutic difficulties and high mortality. Since TBIR is known to be associated with autoimmune diseases such as systemic lupus erythematosus (SLE), glucocorticoids are often used as key immunosuppressive agents. However, glucocorticoids have the potential to exacerbate the pathophysiology of TBIR by worsening insulin sensitivity, which leads to hyperglycemia and muscle wasting. Here, we report a case history of a 66-year-old man who was diagnosed as having TBIR in combination with SLE and Sjögren’s syndrome with marked hyperglycemia, ketosis, and muscle wasting. He was successfully treated with combination therapy of double-filtration plasmapheresis (DFPP) and administration of the anti-CD20 monoclonal antibody rituximab without induction of glucocorticoid therapy while using a sensor-augmented insulin pump (SAP) to prevent hypoglycemia. Remission of diabetes was achieved without severe hypoglycemic events and his circulating insulin receptor antibodies became negative after seven months of initiation of these treatments. Based on the successful clinical courses of this case, our report suggests the possibility of an effective therapeutic regimen with DFPP and rituximab under the condition of the use of an SAP for a patient with TBIR without induction of glucocorticoids.

## Introduction

Type B insulin resistance syndrome (TBIR) is a disorder that was originally reported by Kahn et al. in 1976 as three cases with marked insulin resistance, melanosis nigricans, and the presence of anti-insulin receptor antibodies in blood (1). TBIR is known to be associated with underlying autoimmune diseases such as systemic lupus erythematosus (SLE), Sjögren’s syndrome, various connective tissue diseases, interstitial lung disease, and skin-, blood-, and liver-associated disorders (2). The exact prevalence of TBIR and the involvement of genetic abnormalities remain unknown because of its extreme rarity (3). However, it has been reported that the prognosis is poor if no remission is achieved, resulting in uncontrolled glycemia, muscle wasting and weight loss (4, 5).

A standard treatment for TBIR has not yet been established, although spontaneous remission has been reported in some cases (4). The presence of circulating anti-insulin receptor antibodies underlies the pathogenesis of TBIR, and immunosuppressive therapy is therefore essential for the treatment. Indeed, it has been reported that combination therapy with immunosuppressants such as rituximab, glucocorticoids, cyclophosphamide, and azathioprine contributes to reduction in mortality in patients with TBIR (5). Glucocorticoid is the most commonly used immunosuppressant; however, glucocorticoid can exacerbate insulin resistance, leading to hyperglycemia, which are central to the pathophysiology of TBIR (6). Furthermore, long-term maintenance therapy with glucocorticoids may be needed because most reports have indicated that it takes 3 to 12 months to achieve remission defined as amelioration of hyperglycemia and discontinuation of insulin (5, 7) and even more than 2 years in some cases (8). Glycemic control in the acute phase of TBIR is also important for preventing uncontrolled glycemia-induced complications, muscle wasting, and weight loss. Since it has been reported that insulin-like growth factor 1 (IGF-1) treatment (9) and plasmapheresis (10, 11) were effective for controlling blood glucose levels in patients with TBIR, these treatments may be promising for the acute phase of TBIR until immunosuppressive therapies exert effects. It should be noted that in addition to refractoriness to the treatment for hyperglycemia, hypoglycemia is another important reason why treatment is difficult in patients with TBIR. It has been reported that anti-insulin receptor antibodies can act not only as antagonists at high titers but also as agonists at low titers (3). Therefore, caution is needed for preventing hypoglycemia during the treatment of TBIR, but no method to prevent treatment-induced hypoglycemia with a reduced titer in anti-insulin receptor antibodies has been established. 

Considering the above-described facts, the following points are required in a therapeutic strategy for TBIR: 1) immunosuppressive therapies that can achieve rapid remission without worsening insulin resistance, 2) management for glycemia-induced complications such as acute muscle wasting and weight loss by controlling glycemia, and 3) prevention of both hyperglycemia and hypoglycemia events by monitoring blood glucose. Here, we describe a history of a patient with newly diagnosed TBIR with hyperglycemia, hyperinsulinemia and ketosis who was successfully treated with double-filtration plasmapheresis (DFPP) and administration of rituximab while using a recently developed sensor-augmented pump (SAP), which is a device that combines a continuous insulin infusion pump and real-time continuous glucose monitoring (rtCGM). Compared to simple plasma exchange, DFPP, a semi-selective blood-purification modality derived from plasma exchange, has advantages in selectively removing immunoglobulin fractions, minimizing substitution fluid such as fresh frozen plasma or albumin solution, and reducing cost (12). Since fresh frozen plasma, the major substitution fluid in simple plasma exchange, is known to contain high concentrations of glucose (13), we hypothesized that DFPP would be more suitable for the treatment of TBIR than simple plasma exchange. Notably, there was no need to use glucocorticoid, which can exacerbate insulin resistance, until the TBIR was in remission in this case. Our treatment regimen used in this patient may open the way for the establishment of an effective therapeutic strategy for TBIR.

## Case report

A 66-year-old Japanese man was referred to Sapporo Medical University Hospital for treatment of newly developed TBIR. He had suffered from discoid lupus with leucopenia, hypocomplementemia, positive anti-nuclear antibody and anti-Smith antibody. He was diagnosed with SLE at the age of 48 years and had been stable on only topical therapy for discoid lupus. At the age of 65 years, his regular medical checkup did not reveal any glucose intolerance. However, six months later, he developed both polyuria and thirst and visited his local doctor. He was diagnosed as having diabetes mellitus with blood glucose of 300 mg/dl and HbA1c of 9.1%, and he was admitted to his local hospital. He was initially treated with metformin and multiple daily injections of insulin, but his hyperglycemia did not improve even with a total insulin dose of 120 units/day (insulin degludec of 30 units and insulin aspart of 30 units per each meal). Addition of sulfonylureas and dulaglutide, a weekly glucagon-like peptide 1 (GLP-1) receptor agonist, at 0.75 mg/week also did not improve his hyperglycemia. In addition to marked elevation in fasting serum insulin and C-peptide levels, serum anti-insulin receptor antibody test was positive, leading to the diagnosis of TBIR. He was transferred to our hospital for further treatment of TBIR.

On admission, his body weight was 70.0 kg, body mass index (BMI) was 24.2 kg/m^2^, blood pressure was 85/64 mmHg, pulse rate was 107 bpm, and body temperature was 36.1°C. Since he was suffering from hyperglycemia and anorexia, decreased blood pressure with tachycardia on admission was considered to be due to intravascular dehydration caused by osmotic diuresis. There was no skin rash including melanosis nigricans, erythema spheroids or discoid lupus. Achilles tendon reflex was not decreased, but bilateral lower extremity vibratory sensation was decreased. Results of laboratory tests on admission revealed severe insulin resistance and ketosis: fasting blood glucose level was 225 mg/dl, fasting serum insulin level was 455 μIU/ml, fasting serum C-peptide level was 5.45 ng/ml, and β-hydroxybutyrate level was 1.8 mM (**Table 1**). The activity of SLE was assessed by Systemic Lupus Erythematosus Disease Activity Index-2K (SLEDAI-2K) (14) and it was 4 points for hypocomplementemia (2 points), thrombocytopenia (1 point), and leukopenia (1 point). In addition, serum anti-Sjögren’s-syndrome-related antigen A (anti-SS-A) and B (anti-SS-B) antibodies were positive and the Schirmer test was positive (right 3 mm/5 min, left 1 mm/5 min) together with positive ocular surface staining by fluorescein, indicating a new diagnosis of Sjögren’s syndrome (15). There was no diabetic retinopathy on his eyes. Since pancytopenia was observed, bone marrow aspirations and biopsies were performed and revealed fatty marrow, resulting in a diagnosis of pancytopenia secondary to autoimmune diseases. No malignancy was found in his body.

**Table 1:** Laboratory findings on admission.

<Complete blood count>			<Diabetes-related and Endocrinology measurements>			<Immunological tests>		
WBC	2200	/μL	β-hydroxybutyrate	1.8	µM	CRP	<0.10	mg/dL
Neutrocyte	68	%	Glucose	226	mg/dL	IgG	1843	mg/dL
Lymphocyte	20	%	HbA1c	10.8	%	IgA	435	mg/dL
Monocyte	5	%	Insulin	452.7	µIU/mL	IgM	44	mg/dL
Eosinocyte	5	%	C-peptide	5.45	mg/dL	C3	57	mg/dL
Basocyte	2	%	HOMA-IR	252.6		C4	12	mg/dL
RBC	400	x10^4^/μL	HOMA-β	999	%	CH50	38	/mL
Hb	12.1	g/dL	C-peptide index	2.41		ANA	160	folds
Ht	36.3	%	Anti-GAD antibody	(-)	U/mL	RF	(-)	
Plt	9.8	x10^4^/μL	Anti-IA-2 antibody	(-)	U/mL			
<Biochemistry measurements>			Anti-insulin antibody	(-)	%	Anti-dsDNA antibody	(-)	IU/mL
TP	7.0	g/dL	Anti-insulin receptor antibody	(+)		Anti-SS-A antibody **	16	folds
Alb	3.6	g/dL				Anti-SS-B antibody **	8	folds
T-bil	0.7	mg/dL	TC	145	mg/dL	Anti-CCP antibody	0.7	U/mL
AST	20	IU	TG	40	mg/dL	Anti-Sm antibody **	(-)	folds
ALT	18	IU	HDL-C	47	mg/dL	Anti-RNPantibody **	(-)	folds
ALP*	80	IU	LDL-C	90	mg/dL	Anti-CL IgG antibody	< 2.6	IK/mL
LDH	151	IU	TSH	1.42	μIU/mL	Anti-CL IgM antibody	2.2	IJ/mL
BUN	13	mg/dL	FT3	2.4	pg/mL	Anti-β2GPl IgG antibody	12.6	IJ/mL
Cr	0.5	mg/dL	FT4	1.22	ng/dL	Anti-β2GPl IgM antibody	< 1.1	IJ/mL
UA	4.0	mg/dL	ACTH	13.4	pg/mL	PA-IgG	48.6	ng/10^7^cells
Na	137	mEq/L	Cortisol	9.76	µg/dL	Lupus anticoagulant test	(-)	
K	3.8	mEq/L				Urine anti-HP antibody	(+)	

WBC, white blood cell; RBC, red blood cell; Hb, hemoglobin; Ht, hematocrit; Plt, platelet; TP, total protein; Alb, albumin; T-bil, total bilirubin; AST, aspartate aminotransferase; ALT, alanine aminotransferase; ALP, alkaline phosphatase; LDH, lactate dehydrogenase; BUN, blood urea nitrogen; Cr, creatinine; UA, uric acid; HbA1c, hemoglobin A1c; HOMA-IR, Homeostatic Model Assessment - Insulin Resistance; HOMA-β, Homeostasis Model Assessment for β-cell function; Anti-GAD antibody, anti glutamic acid decarboxylase antibody; Anti-IA-2 antibody, anti islet antigen 2 antibody; TC, total cholesterol; TG, triglycerides; HDL-C, high-density lipoprotein cholesterol; LDL-C, low-density lipoprotein cholesterol-cholesterol; TSH, thyroid-stimulating hormone; FT3, free thyroxine 3; FT4, free thyroxine 4; ACTH, adrenocorticotropic hormone; CRP, C-reactive protein; ANA, antinuclear antibody; RF, rheumatoid factor; Anti-dsDNA antibody, double-stranded DNA antibody; Anti-SS-A antibody, anti Sjögren’s-syndrome-related antigen A; Anti-SS-B antibody, anti Sjögren’s-syndrome-related antigen B; Anti-CCP antibody, anti-cyclic citrullinated peptide antibody; Anti-RNP antibody, anti-ribonucleoprotein antibody; Anti-Sm antibody, anti-Smith antibody; Anti-CL antibody, anti-caldiolipin antibody; Anti-β2GPl antibody, anti-β2 glycoprotein l antibody; PA-IgG, platelet-associated IgG; Urine anti-HP antibody, urine anti-Helicobacter pylori antibody.*, Tested by Japan Society of Clinical Chemistry method. **, Tested by Ouchterlony method.

**Figure 1:**
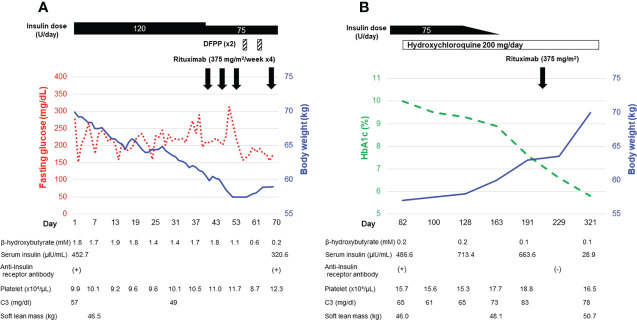
Clinical course of the patient during hospitalization **(A)** and in the outpatient setting **(B)**.

**Figure 2:**
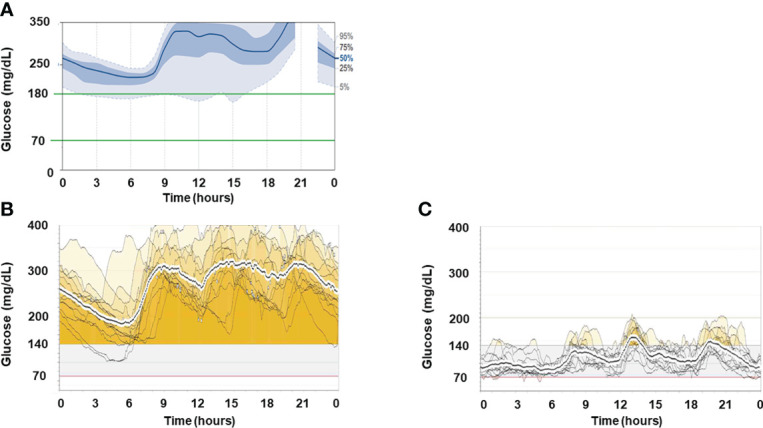
Representative glucose profiles of CGM recordings. **(A)** Representative ambulatory glucose profile recorded by isCGM before treatment. **(B)** Two-week glucose profiles recorded by rtCGM one month after the initiation of treatment. **(C)** Two-week glucose profiles recorded by rtCGM nine months after the initiation of treatment.

His clinical course after admission is shown in **Figure 1A**. Results of intermittently-scanned CGM (isCGM, FreeStyle Libre, Abbott Diabetes Care Inc., CA, U.S.A.) revealed severe hyperglycemia throughout the day without evidence of hypoglycemia (**Figure 2A**). In addition to persistent ketosis, his body weight was progressively reduced, suggesting catabolic state. Therefore, we decided to perform combination therapy with DFPP (twice a week) and infusion of the anti-CD20 antibody rituximab (375 mg/m^2^, once weekly for 4 weeks) as previously reported (16), followed by administration of hydroxychloroquine for reducing SLE activity. Furthermore, an SAP (MiniMed 640G system, Medtronic, Dublin, Ireland), which can detect rapid decline in interstitial glucose levels, was introduced instead of isCGM (**Figure 2B**) due to early detection of hypoglycemic event. Following these combined treatment strategies, hyperglycemia and ketosis improved, and he was discharged on his 71st admission day. Thereafter, his HbA1c gradually improved and no severe hypoglycemia occurred due to glycemic monitoring and fine-tuning of insulin dosage using an SAP. Additional rituximab administration (375 mg/m^2^/week, 1 time) was performed six months after initial infusion. Anti-insulin receptor antibodies became negative after seven months of initiation of the therapy. Representative glucose profiling nine months after these treatments is shown in **Figure 2C**. Soft lean mass evaluated by bioelectrical impedance analysis was increased from 46.5 kg at baseline to 50.7 kg after his anti-insulin receptor antibodies became negative, suggesting an improvement in the catabolic state (**Figure 1B**). Platelets counts and hypocomplementemia were also recovered with an improvement of glycemic control and SLEDAI-2K became 1 point seven months after the initiation of therapy (**Figure 1B**). There were no remarkable adverse events including infusion reaction, opportunistic infections, severe infections, gastrointestinal symptoms, retinopathy and hemorrhagic events throughout his clinical course.

## Discussion

There are two salient points in this report: 1) A combination therapy including DFPP and rituximab, followed by hydroxychloroquine administration, led to successful remission of TBIR without use of glucocorticoid therapy in a TBIR patient complicated with SLE and Sjögren’s syndrome. 2) SAP was useful to the monitoring and control of glucose levels during aggressive TBIR therapy.

Since the patient had progressive weight loss and muscle wasting, DFPP was used as an acute-phase therapy to prevent catabolism by restoring insulin sensitivity *via* removal of anti-insulin receptor antibodies. Actually, DFPP has been applied to various conditions including autoimmune diseases and organ transplantations (12, 17, 18). Since TBIR has been reported to be an autoimmune disorder caused by polyclonal autoantibodies (3), mechanical removal of such autoantibodies using DFPP is a reasonable therapeutic approach. Indeed, some previous case reports have shown rapid improvement in hyperglycemia after plasmapheresis, suggesting the effectiveness in this acute phase treatment (10, 11). Since albumin solution, rather than fresh frozen plasma containing high concentrations of glucose (13), is usually used as the substitution fluid in DFPP, DFPP might be an optimal plasmapheresis for the treatment of TBIR, which is characterized by severe insulin resistance. In contrast, we acknowledge that DFPP has a possible disadvantage in loss of high molecular weight coagulation factors that can lead to hemorrhagic events (19). To avoid this side effect of DFPP, we took the following actions in the present case: 1) levels of serum fibrinogen, one of the high molecular weight coagulation factors, were measured before DFPP and 2) only two DFPP sessions were performed. Fortunately, in the present case, the levels of fibrinogen never reached below 100 mg/dl, which we had set as a criterion for discontinuation of DFPP. In addition, no hemorrhagic events were found throughout his clinical course. Thus, monitoring serum fibrinogen levels and performing fewer DFPP sessions may lead to early detection or prevention of coagulation factor depletion in DFPP, thereby preventing hemorrhagic events.

One interesting aspect of this case is that blood β-hydroxybutyrate became negative despite persistent hyperglycemia after treatment with DFPP (**Figure 1**). Although the precise reason for the differential effects of DFPP on blood glucose and ketone metabolism in TBIR remains unknown, insulin signaling is known to be different in organs or types of cells including adipocytes, hepatocytes, and myocytes (20) and it is possible that the temporary decrease in anti-insulin receptor antibodies caused by DFPP preferentially improved insulin signaling only in adipocytes or hepatocytes. It should also be noted that DFPP does not suppress the production of anti-insulin receptor antibodies from abnormal B cells; therefore, its efficacy is temporal. Taken together, DFPP may be specifically effective for patients with TBIR who are suffering from extremely high insulin resistance and progressive catabolism.

As an alternative to DFPP, treatment with IGF-1 has been reported to be an option for acute treatment of TBIR (9). IGF-1 can promote glucose uptake into cells *via* binding to IGF receptors or insulin receptors. Thus, administration of recombinant IGF-1 could theoretically activate intracellular glucose uptake pathways *via* an alternative pathway rather than insulin signaling that is inhibited by the anti-insulin receptor antibody. However, a recent report indicated that the effectiveness of IGF-1 for TBIR may be limited (21). This limitation is due to the possibility that anti-insulin receptor antibodies also have an affinity for the IGF-1 receptor (22) or that IGF-1 itself is not effective for removing the anti-insulin receptor antibodies that are the causative molecules of TBIR. Of course, IGF-1 treatment may contribute to better outcomes for TBIR by improving hyperglycemia, but we thought that DFPP was likely to surpass the beneficial effects of administration of recombinant IGF-1, and we chose DFPP as the treatment of choice for the acute phase in the present case.

One of the important factors contributing to the successful outcome of this case was that the patient achieved remission with negative anti-insulin receptor antibody by rituximab without glucocorticoid therapy. The effects of rituximab for TBIR are controversial (7, 23, 24) and rituximab was administered with concomitant use of glucocorticoids in previous cases. In general, rituximab takes several weeks after administration to exert its effect for suppression of antibody production. Rapid remission induction is important in autoimmune diseases such as anti-neutrophil cytoplasmic antibody-associated vasculitis or immune thrombocytopenic purpura, and concomitant use of glucocorticoids and rituximab is needed to achieve rapid remission induction. In our case, we used DFPP instead of IGF-1 or glucocorticoids to avoid acute phase complications and it worked well in our case. Other immunosuppressants such as cyclophosphamide have also been reported to be effective for TBIR, but glucocorticoids were used in combination in those cases (7). In this case, administration of cyclophosphamide was avoided due to pancytopenia. It is still unclear if hydroxychloroquine improved TBIR as an immunomodulatory drug; however, hydroxychloroquine was added as a standard-of-care treatment for SLE.

Autoantigens that may trigger the development of TBIR also remain unknown. Imai et al. previously reported the association between TBIR and *Helicobacter pylori* infection (25). Indeed, the patient’s urine anti-*Helicobacter pylori* antibody was positive on admission (**Table 1**), although he achieved remission without *Helicobacter pylori* eradication treatment. Additionally, systemic autoimmune diseases such as SLE and Sjögren’s syndrome are known to be associated with TBIR. In the present case as well, the patient was complicated with SLE and Sjögren’s syndrome. The disease severity of SLE was mild at the onset of TBIR, but platelet counts and hypocomplementemia recovered along with a decrease in the titer of anti-insulin receptor antibodies, suggesting that TBIR may be associated with concomitant autoimmune diseases. Since TBIR is an extremely rare disease, its detailed etiology remains unknown. Further studies will be needed to clarify the detailed pathogenesis of TBIR.

The pattern of glycemic variability in TBIR has been reported to include only hyperglycemia and a mixture of hyperglycemia and hypoglycemia, although a recent report recommended that a case showing hypoglycemia alone should be excluded from the diagnosis of TBIR (26). Hyperinsulinemia induced by increased endogenous insulin secretion in response to severe insulin resistance is elicited in most cases of TBIR. Thus, the development of significant hypoglycemia, whether treatment-related or not, requires careful attention. Therefore, an SAP may be the best tool for both treating TBIR and monitoring blood glucose to prevent hypoglycemia as it allows adjustment of insulin dosing while monitoring blood glucose with rtCGM. In this case, because of increased endogenous insulin secretion due to consistent severe insulin resistance, the dose of insulin infusion was not increased according to blood glucose levels, and a strategy that prioritizes the avoidance of hypoglycemia was chosen. Whether increasing doses of insulin infusion or using a closed-loop insulin delivery system (27) such as a system that optimizes insulin doses according to blood glucose would result in better outcomes in TBIR is unknown and requires further studies.

In summary, we described a case history of a patient with TBIR who presented hyperglycemia, hyperinsulinemia, and ketosis and who was successfully treated with an SAP in combination with DFPP and rituximab. Our therapeutic approach may provide an effective and novel treatment strategy that could become a new insight for the care of TBIR.

## Ethics statements

Ethical review and approval was not required because this is not a clinical study but a case report in accordance with the local legislation and institutional requirements. Written informed consent was obtained from the patient for the publication of any potentially identifiable images or data included in this article.

## Author contributions

AO, MK, and TS designed the structure of the manuscript and wrote the original manuscript. AO and TS prepared figures and tables. CA, SB, HK, and KM analyzed the patient data and revised the manuscript. TomY and TosY supervised the patient’s clinical course and revised the manuscript. All authors contributed to the article and approved the submitted version.

## Funding

This work was supported by Education and Research Grants in 2021-2022 from Sapporo Medical University.

## Acknowledgments

The authors thank all physicians and health care professionals who were involved in the treatment of this case. The authors would like to thank Dr. Naoyuki Kitao (Japan Community Healthcare Organization Hokkaido Hospital) for his cooperation in our investigation of the patient’s clinical course. The authors acknowledge S.E.S. Translation and Proofreading Services for editing and proofreading this manuscript.

## Conflict of interest

TS: Honoraria (lecture fee) from Abbott Japan LLC., Novo Nordisk Pharma Ltd.

The remaining authors declare that the research was conducted in the absence of any commercial or financial relationships that could be construed as a potential conflict of interest.

## Publisher’s note

All claims expressed in this article are solely those of the authors and do not necessarily represent those of their affiliated organizations, or those of the publisher, the editors and the reviewers. Any product that may be evaluated in this article, or claim that may be made by its manufacturer, is not guaranteed or endorsed by the publisher.
